# Does Dominant Somatotype Differentiate Performance of Jumping and Sprinting Variables in Young Healthy Adults?

**DOI:** 10.3390/ijerph191911873

**Published:** 2022-09-20

**Authors:** Fahri Safa Cinarli, Hakan Buyukcelebi, Ozcan Esen, Magdalena Barasinska, Ladislav Cepicka, Tomasz Gabrys, Umut Nalbant, Raci Karayigit

**Affiliations:** 1 Department of Coaching Education, Faculty of Sport Sciences, Inonu University, Malatya 44000, Turkey; 2 Department of Sport, Exercise and Rehabilitation, Northumbria University, Newcastle upon Tyne NE1 8ST, UK; 3 Department of Health Sciences, Jan Dlugosz University, 42-200 Czestochowa, Poland; 4 Sport Centrum, Faculty of Pedagogy, University of West Bohemia, 30100 Pilsen, Czech Republic; 5 Department of Exercise and Sport Sciences, Faculty of Health Sciences, Eastern Mediterranean University, Famagusta 99628, Turkey; 6 Department of Coaching Education, Faculty of Sport Sciences, Ankara University, Ankara 06830, Turkey

**Keywords:** anthropometry, explosive movement, peak power, somatotype

## Abstract

The relationship between an athlete’s somatotype three-numeral rating and his or her athletic performance is well known. However, a direct effect of the different dominant somatotype on jumping and sprinting variables has not yet been reported. The aim of this study was to investigate the effects of dominant somatotype on sport-specific explosive variables. One hundred and twelve physically active young adults (mean ± standard deviation age: 21.82 ± 3.18 years) were somatotype-rated using the Heath–Carter method. Participants were classified as balanced ectomorph, balanced mesomorph, central, mesomorph-endomorph, and mesomorphic ectomorph. Vertical jump and linear sprint tests were performed to measure peak lower body performance and sprint variables (time, speed, and momentum), respectively. The analysis revealed that balanced mesomorph had significantly higher vertical jump (effect size (ES) = 1.10, *p* = 0.005) and power to body mass (ES = 1.04, *p* = 0.023) than mesomorph-endomorph. In addition, balanced mesomorph showed significantly superior performance in 30-m sprint time and velocity than central and mesomorph-endomorph (ES range = 0.93–1, *p* < 0.05). Finally, balanced ectomorph (ES = 1.12, *p* = 0.009) and mesomorphic ectomorph (ES = 1.10, *p* = 0.017) were lower in sprint momentum compared to balanced mesomorphs. In conclusion, this study has shown the importance of the interaction between subtypes and athletic performance. The knowledge gained may be important in identifying those who tend to perform well in sports with explosive power and in prescribing training programs.

## 1. Introduction

Somatotype assessment is used to classify the physique using scales that relate to body shape and composition and to assess adiposity, musculo-skeletal robustness, and linearity or slenderness [1] Somatotype is an important classification system in which the body is classified according to anthropometric measurement values in general and the appearance can be interpreted as quantitative data [2]. The somatotype can be identified by assigning a three-numeral rating representing endomorphy, mesomorphy, and ectomorphy [3].

It is well known that differences in somatotype have a direct impact on athletic performance and are one of the most important criteria in the selection of athletes [4,5]. The reason why 65% of the variance in the results obtained in physical fitness tests in adult athletes is explained by somatotype, which reveals the rational basis of this situation [6]. One study found that morphological typology interacted more strongly with strength than body fat and physical activity [7]. It is therefore considered that studies investigating athletic performance should also take into account differences in somatotype, which can have a significant impact on the results. However, many studies in the literature examine correlation or regression analyses between the three-digit classification of somatotype (endo-meso-ecto) and performance outcomes [8,9]. On the other hand, groups with different dominant somatotypes should be compared to better understand the effects of differences in somatotype on athletic performance. In this way, it is possible to determine the body type that can demonstrate optimal success with a particular motor skill. 

Explosive movement performance is a biomotor skill that directly influences performance in many sports [10]. The ability to generate maximum force or change position in a short time is an important advantage for athletes. Somatotype has been found to influence anaerobic performance, especially in activities that require strength, where mesomorphic levels have an advantage [11,12]. When examining somatotype and explosive force values, it was found that an increased endomorphy value had a negative effect on vertical jump performance, which assessed the change in position of the centre of mass against gravity, while an increased ectomorphy value had a positive effect on vertical jump and sprint performance [13,14]. The rational reason for the fact that those with high levels of mesomorphy have an advantage in vertical jump and sprint performance could be the positive relationship between muscle mass and mesomorphy [15]. Since endomorphy stands for fatness, fat-free mass is generally lower in endomorphs than in mesomorphs. In terms of power output, especially in parameters such as the vertical jump and sprint, where maximum power is expected in a short time, muscle mass has a direct effect on the outcome [16]. Thus, differences in somatotype were found to have a significant impact on vertical jump and linear sprint performance [17,18]. In principle, however, it is possible to predict how levels of obesity, muscularity, and weakness may affect vertical jump and sprint performance. However, identifying the differences between subtypes and determining the effect sizes of these subtypes on the measured parameters may provide an opportunity for a deeper understanding of the role and importance of the dominant somatotype in athletic performance.

It is known that the somatotype does not consist of only three digits, and it is assumed that the effects of the subtypes should be investigated in order to interpret the effects on the variables vertical jump and linear sprint more precisely. Therefore, the aim of this study is to examine the effects of somatotypes determined in young adults on vertical jump and linear running performance variables. We hypothesized that dominant somatotype would have a significant effect on selected variables. 

## 2. Materials and Methods

### 2.1. Subjects

Data were collected from students at the faculty of sports sciences. One hundred and twelve physically active young adults (mean ± standard deviation (SD) age: 21.82 ± 3.18 years; height: 1.73 ± 0.08 m; body mass: 63.3 ± 10.49 kg) were recruited to the study, which included 77 men (mean ± SD age: 21.92 ± 3.59 years; height: 1.77 ± 0.06 m; body mass: 67.33 ± 8.08 kg) and 35 women (age: 21.60 ± 2.06 years; height: 1.64 ± 0.05 m; body mass: 54.42 ± 9.78 kg). Inclusion criteria for all subjects were that none of the subjects had a history of musculoskeletal, neurological, or orthopedic disease that might have affected their ability to perform the vertical jump and linear sprint tests. The exclusion criterion was groups with a dominant somatotype of less than 20 people in total. This was to ensure that the groups to be compared were close in number. This study was approved by the Ethics Committee of Inonu University Clinical Research (Protocol Number: 2022/3557). The study was conducted in accordance with the principles of the Declaration of Helsinki. After the subjects were informed about the study, informed consent forms were signed. 

### 2.2. Experimental Design

The study was conducted over a total of two days. On the first day, anthropometric measurements were taken, and the somatotypes determined. Somatotype classification included at least 20 participants for reliability of statistically significant effect. Body types identified below 20 were excluded from the study. The number of groups with at least 20 individuals whose somatotypes were determined was set at five. Then, the sample size was determined by a priori power analysis with G-Power (Version 3.1, Dusseldorf, Germany). An effect size of 0.4 was considered a reasonable and conservative point for determining the sample size. Type I error (α) was 0.05 and power (1-β) was 0.80 with analysis of variance (fixed effects, omnibus and one-way test). The model indicated a minimum total sample size of 80 subjects, but taking into account a 20% missing participant rate, we decided on a total number of at least 100 participants. As mentioned earlier, participants were classified as balanced ectomorph (n = 25), balanced mesomorph (n = 22), central (n = 24), mesomorph-endomorph (n = 21), and mesomorphic ectomorph (n = 20). On the second day, athletic performance tests were conducted with the defined groups. First the vertical jump was performed, then the linear sprint test. Vertical jumping and linear running were both performed at the Faculty of Sport Science Sports Complex on a rubber surface, on a day set by an experienced coach. Before performing the athletic performance tests, the subjects warmed up with 5 min of jogging at self-paced speed, followed by 2 min of dynamic stretching. In addition, participants were asked to experiment with 50% effort before the formal measurements to ensure familiarity with the test. The tests took place between 10:00 and 11:00 a.m. and the subjects did not eat in the 2–3 h before the test. The independent variables were selected as dominant somatotypes and the dependent variables as athletic performance parameters.

### 2.3. Anthropometric Measurements and Somatotype Classification

**Figure 1:**
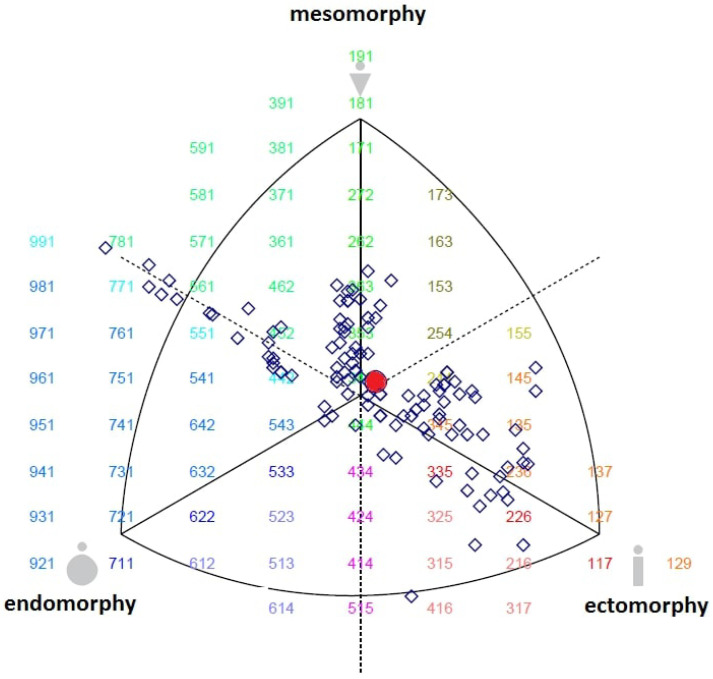
Somatotype profile distribution of young adults (n = 112). The squares are the individual somatotypes and the red circle is the mean profile.

Anthropometric profiles of the subjects were measured according to the protocols of the International Society for the Development of Kinanthropometry with a technical error of measurement less or equal to 1% [19]. All measurement procedures were performed with minimal clothing and no shoes. Participants’ height was measured with a portable stadiometer (Seca Ltd., Bonn, Germany) with an accuracy of 0.1 cm, with the head in the Frankfort plane while the body was upright, and the weight was evenly distributed on both legs. Body mass was measured using a bioelectrical impedance with a capacity of 270 kg and a sensitivity of 100 g (Tanita SC -330S, Amsterdam, The Netherlands). The triceps, subscapular, suprailiacus, and medial calf skinfolds (using a caliper), upper arm girth and medial calf girth (using a nonstretchable tape), and bi-epicondylar humerus and bi-epicondylar femur breadth (using a bicondylar caliper) were measured for each participant. The data from the anthropometric assessments were used to calculate the somatotype values according to the Heath-Carter method [3]. The somatotype calculations and the position in the somatoplot were performed using the Somatotype 1.2.5 software (Figure 1).

### 2.4. Jump Test Procedure

The countermovement jump was used to examine vertical jump (VJ) height. Study participants were asked to stand with both feet on a contact mat (Fusion Sport Smart Jump Mat, Fusion Sport, Australia). Subjects were instructed to keep their hands on their hips before and during the jump (to control arm use). The correct trial was when the participant had extended his knee joints during the flight and the first landing contact. There was a 2-min break between jumps. The highest jump (cm) from three maximal trials was selected for data analysis [20].

### 2.5. Determining the Absolute and Relative Peak Power

These variables were calculated using the highest jump achieved in 3 repetitions of the VJ. Anaerobic power measured in watts (PAPw) was calculated by the Sayers equation using the following formula: PAPw = 60.7 × jump height (cm) + 45.3 × body mass (kg)–2055 [21]. Power to body mass ratio (P:BM) was derived using the equation: P:BM = PAPw/body mass (kg) [22].

### 2.6. Sprint Test Procedure

Participants’ running speed was assessed by sprinting 30 m using dual-beam electronic timing devices (Fusion Sport Smart Speed; Fusion Sport, Australia). Gates were placed 30 m from a predetermined starting point. Subjects were instructed to run the 30 m distance from a standing position as fast as possible. The participants started each sprint 30 cm behind the starting line to trigger the first one. There was a 2-min break between sprints. Speed was measured to the nearest 0.01 s, with the fastest result from three trials used as the speed value [23].

### 2.7. Determining the Velocity and Sprint Momentum

These variables were calculated using the best time achieved in 3 repetitions of the 30-metre sprint. Sprint velocity was determined by dividing the distance (30 m) by the time (speed = distance (m)/sprint time (s)). This was then multiplied by the subjects’ body mass in kg, which gave the momentum (sprint momentum = body mass (kg) × velocity (m/s)) [24].

### 2.8. Statistical Analysis

Statistical analyses were processed using the Statistical Package for Social Sciences (Version 24; IBM Corporation, NY, USA). Data normality was assessed using the Kolmogorov–Smirnov test. Descriptive data (mean ± SD; 95% confidence interval (CI)) were calculated for participants. A one-way analysis of variance (ANOVA) was used to evaluate somatotype group differences, with significance set at *p* < 0.05 (balanced ectomorph, balanced mesomorph, central, mesomorph-endomorph and mesomorphic ectomorph). Eta squared (η^2^) was calculated as a measure of effect size for each ANOVA. The thresholds for small, medium, and large effects were 0.01, 0.06 and 0.14, respectively [25]. Multiple pairwise comparisons were determined by the Bonferroni post hoc test when the variances were equal and by the Games–Howell test when they were not equal. Effect sizes (d) were also calculated for the between-group comparisons for somatotypes, where the difference between the mean values was divided by the pooled SD [25]. In accordance with Hopkins, a less than 0.2 was considered a trivial effect; 0.2–0.6 a small effect; 0.6–1.2 a moderate effect; 1.2–2.0 a large effect; 2.0–4.0 a very large effect; and 4.0 and above an extremely large effect [26].

## 3. Results

The data for the subjects are shown in Table 1. Mean (± SD, minimum, and maximum with 95% CI) somatotype for the subjects (n = 112) was endomorphy 3.03 (± 1.08), mesomorphy 3.55 (± 1.13), ectomorphy 3.44 (± 1.37). Subjects’ athletic performance characteristics for each test are provided in Table 2.

Participants were classified as balanced ectomorph (n = 25), balanced mesomorph (n = 22), central (n = 24), mesomorph-endomorph (n = 21), and mesomorphic ectomorph (n = 20). Group demographics and comparisons are shown in Table 3. The one-way analysis of demographic differences revealed a significant difference between the somatotype groups in weight (F(4,107) = 11.198; *p* < 0.001, η^2^ = 0.29), but there was no significant difference between the age (F(4,107) = 2.000; *p* = 0.100, η^2^ = 0.07) and height (F(4,107) = 1.556; *p* = 0.191, η^2^ = 0.05) of the groups. The analysis showed that balanced ectomorph (ES range = 1.18–1.46, *p* < 0.01) and mesomorphic ectomorph (ES range = 1.27–1.53, *p* < 0.01) had significantly lower body mass than balanced mesomorph and mesomorph endomorph. In addition, mesomorph endomorph showed significantly higher in the body mass compared with central (ES = 0.87, *p* = 0.025). Three-item somatotype scores showed significant differences between groups in endomorphy (F(4,107) = 95,491, η^2^ = 0.78), mesomorphy (F(4,107) = 68,615, η^2^ = 0.71), and ectomorphy (F(4,107) = 93,396, η^2^ = 0.77) scores (*p* < 0.001).

The athletic performance variables for the different somatotype groups were presented in Table 4. One-way analysis of field tests revealed a significant difference between somatotype groups for sprint time (F(4,107) = 3.117; *p* = 0.018, η^2^ = 0.10), vertical jump (F(4,107) = 4.043; *p* = 0.004, η^2^ = 0.13), p:BM (F(4,107) = 3.134; *p* = 0.018, η^2^ = 0.10), sprint momentum (F(4,107) = 4.657; *p* = 0.002, η^2^ = 0.14), and sprint speed (F(4,107) = 3.306; *p* = 0.014, η^2^ = 0.11), except for PAPw (F(4,107) = 1.915; *p* = 0.113, η^2^ = 0.06).

**Figure 2:**
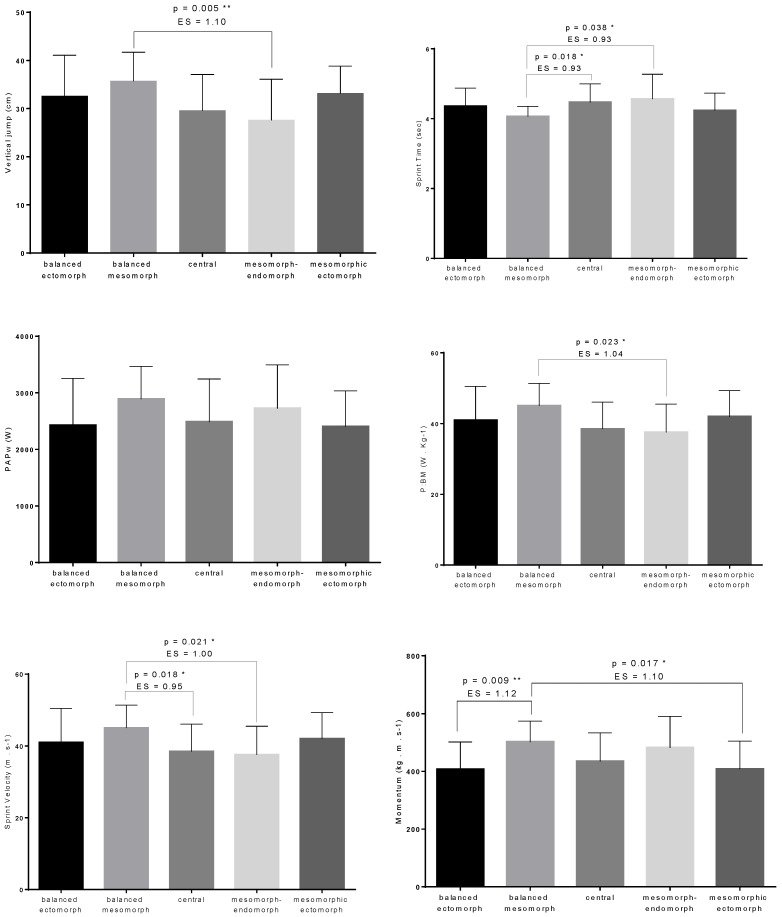
Multiple pairwise comparisons for the jumping and sprinting variables in participants by dominant somatotype (* = *p* < 0.05; ** = *p* < 0.01).

Multiple pairwise comparisons are presented in Figure 2. The analysis revealed that balanced mesomorph had significantly higher VJ (ES = 1.10, *p* = 0.005) and power to body mass (ES = 1.04, *p* = 0.023) than mesomorph-endomorph. In addition, balanced mesomorph showed significantly superior performance in 30-m sprint time and velocity than central and mesomorph-endomorph (ES range = 0.93–1, *p* < 0.05). Finally, balanced ectomorph (ES = 1.12, *p* = 0.009) and mesomorphic ectomorph (ES = 1.10, *p* = 0.017) were lower in the sprint momentum compared with balanced mesomorph. All significant differences had moderate effect sizes.

## 4. Discussion

This study investigated the effects that different dominant somatotype may have had on the vertical jump and sprint variables. The results indicated that balanced mesomorph tended to perform higher in power to body mass and vertical jump assessments when compared with mesomorph-endomorph. Moreover, central and mesomorph-endomorph showed statistically significant lower performance in linear sprint performance variables. Furthermore, our results show that the improvements in momentum were attributable to increases in body mass, as balanced ectomorphs and mesomorphic ectomorph had significantly lower sprint momentum than balanced mesomorphs. 

In our study, it was found that only the mesomorph-endomorph group with the greatest body weight showed significantly lower performance in the vertical jump compared to the balanced mesomorphs in the performance data. However, there was no significant difference between these two groups in peak anaerobic power. There was, however, a statistically significant difference in the power to body mass ratio with the effect of body weight. It can be said that the increase in the endomorphy component expressing fatness has a negative effect on the ability to shift the vertical axis of mass. In handball players, somatotypes were determined in relation to their playing positions, and it was found that the vertical jump performance of goalkeepers and pivot players with the predominant endomorph was lower than that of the others [27]. In support of this view, a significant negative correlation was found between the endomorphy component and leg explosive strength obtained from the vertical jump test [28]. Another study found a negative correlation between body fat percentage and vertical jump performance (*r* = −0.57, *p* < 0.01) [29]. The findings in the study suggest that the increase in body mass and fatness, together with the susceptibility to endomorphy, is a significant disadvantage when jumping vertically against gravity [30]. On the other hand, some studies show that players in volleyball, where the vertical jump is extremely important, are balanced mesomorphs [31,32], and other studies have found that the mesomorphic or ectomorphic component dominates depending on the playing position [33]. In our study, the highest performance results in terms of power to body mass ratio and vertical jump height were obtained by balanced mesomorphic and ectomorphic groups. This finding can be interpreted as a similar result to many studies regarding balanced mesomorphy and ectomorphy.

A significant positive correlation was found between the mesomorphic component and anaerobic performance parameters [11]. In our study, moderate and large effect sizes were found between groups in relation to sprint variables (η^2^ range = 0.10–0.14, *p* < 0.05). In addition, the group with the balanced mesomorph was clearly superior in sprint speed and sprint time. This is due to the fact that balanced mesomorphic individuals have an advantage in terms of skeletal muscle mass. Muscle mass is an important factor in the ability to generate maximal force per unit time. In a study investigating the effects of somatotype on sprint start and acceleration performance, it was found that more muscular senior athletes had better running acceleration than junior athletes [34]. Thus, when explosive, strength-based sports such as football are studied, it is found that athletes generally have a balanced mesomorphic body type [35]. It is also noted that the fact that sprinters have less fat mass is a rational effect in terms of their superiority in linear sprint speed and time [36]. The findings in the study in favor of balanced mesomorphs in terms of sprint speed and time are consistent with the literature. 

It has been mentioned that sprint momentum is a much more effective measurement tool than sprint speed in assessing linear sprint performance [24]. There is no study in the literature that examines the influence of the body types we have identified on sprint momentum. Therefore, it will be difficult to compare the somatotypes identified in this study with the literature. However, an evaluation was made in relation to the rational effect of body weight on momentum. In our study, it was found that the balanced ectomorph and mesomorphic ectomorph groups, which had a higher ectomorphic component, were disadvantaged in momentum. One of the most interesting findings of the study was that when the sprint time and sprint speed of these groups were examined, there was no significant difference compared to the balanced mesomorphic groups, but significant differences were found observed in the momentum data. The similarity in sprint speed but the significant difference in sprint momentum between somatotype groups are consistent with previous studies [37,38]. When calculating sprint momentum, the possibility that differences in somatotype directly affect momentum was strengthened by considering sprint speed along with body mass. In our study, the ectomorphic participants (balanced ectomorph and mesomorphic ectomorph) had lower body weight than other dominant groups. While no significant difference is found in the literature between the sprint times of professional first and second division rugby players, it has been observed that the momentum scores of first division players with an average body weight of 7% are significantly higher [39]. In another study, it was mentioned that the increase in body mass can have a positive effect on performance in sprinting [24]. In our study, the fact that the highest sprint momentum performance after the balanced mesomorphs belongs to the group of mesomorphic endomorphs also coincides with the rational explanation of this situation. 

In our study, there are some limitations regarding the selected sample and performance tests. First, although vertical and linear tests were chosen in relation to jumping and running, the effects of horizontal jumps and changes of direction, which are important for many sports, could also be studied. Secondly, the results are aimed at the general population, as the group participating in the study has no professional experience in any sport. Therefore, future studies can examine athletes who play professional sports as much as possible and classifications can be made according to their playing positions. Nevertheless, extremely valuable insights into dominant somatotype differences were gained in our study.

## 5. Conclusions

These results suggest that when observing jump and sprint variables of different somatotype groups, the predominant somatotype may be much more important than the triple classification. Pairwise comparison analyses indicated that balanced mesomorph was the best component of somatotype to show highest performance on lower body strength and linear sprint variables. These data suggest that different dominant somatotypes have an effect on explosive strength, which should not be disregarded. Furthermore, it can be said that the inclusion of body mass in the assessment can provide a more objective means of analysis by using different formulas instead of a single figure when assessing athletic performance. Since we did not examine professional athletes in any sport in our study, we did not have the opportunity to examine them according to their positions. However, the literature suggests that positional differences should be considered as an important factor in studies along these lines. The findings from this study have important implications for strength and conditioning coaches who work with the talent selection process and plan training. 

## Figures and Tables

**Table 1:** Descriptive statistics (mean ± SD; 95% CI) for the demographic variables and somatotype components in young adults (n = 112).

Variables	Mean ± SD	Minimum	Maximum	95% CI
Age (y)	21.82 ± 3.18	19	25	21.23–22.42
Height (m)	1.73 ± 0.08	1.55	1.93	1.71–1.74
Body mass (kg)	63.3 ± 10.49	39.5	89.5	61.33–65.26
Skinfolds (mm)				
Triceps	11.57 ± 4.89	4.3	25	10.66–12.49
Supscapula	12.28 ± 4.52	6	32	11.44–13.13
Suprailiacus	7.06 ± 3.17	2.9	19	6.47–7.66
Medial calf	9.36 ± 4.35	2	26	8.54–10.17
Girth (cm)				
Upper arm	29.6 ± 3.73	22	37	28.91–30.30
Medial calf	37.81 ± 2.90	26	42.7	32.12–43.49
Breadth (cm)				
Humerus	6.3 ± 0.49	5.5	7.6	6.21–6.4
Femur	9.02 ± 0.84	6.1	10.7	8.87–9.18
Endomorphy	3.03 ± 1.08	1.3	6.9	2.83–3.23
Mesomorphy	3.55 ± 1.13	0.2	6.8	3.34–3.76
Ectomorphy	3.44 ± 1.37	0.2	6.2	3.18–3.7

**Table 2:** Descriptive statistics (mean ± SD; 95% CI) for the jumping and sprinting variables in young adults (n = 112).

Variables	Mean ± SD	Minimum	Maximum	95% CI
VJ (cm)	31.74 ± 7.76	15.8	53.58	30.28–33.19
PAPw (W)	2583.04 ± 735.24	937.33	3994.82	2445.37–2720.71
P:BM (W·kg^−1^)	40.79 ± 8.14	22.97	65.76	39.26–42.31
ST (s)	4.34 ± 0.54	3.74	6.69	4.24–4.44
SV (m·s^−1^)	7 ± 0.76	4.48	8.02	6.85–7.14
Momentum (kg·m·s^−1^)	446.47 ± 100.4	234.65	653.28	427.67–465.27

VJ = vertical jump; PAPw = peak anaerobic power measured in watts; P:BM = power-to-body mass ratio; ST = sprint time; SV = sprint velocity.

**Table 3:** Descriptive statistics (mean ± SD) for the demographic variables and somatotype characteristics in participants by dominant somatotype.

Variables	Balanced Ectomorph (n = 25)	Balanced Mesomorph (n = 22)	Central (n = 24)	Mesomorph-Endomorph (n = 21)	Mesomorphic Ectomorph (n = 20)	F	*p*	η^2^
Age (year)	22.08 ± 1.6	21.22 ± 1.65	20.95 ± 1.45	21.76 ± 1.7	21.75 ± 1.33	2.000	0.100	0.07
Height (m)	1.76 ± 0.10	1.73 ± 0.07	1.73 ± 0.07	1.7 ± 0.07	1.73 ± 0.08	1.556	0.191	0.05
Weight (kg)	57.5 ± 8.38	67.66 ± 8.29	63.41 ± 8.42	71.71 ± 10.5	56.43 ± 9.33	11.198	<0.001 **	0.29
Endomorphy	2.40 ± 0.57	2.75 ± 0.32	3.29 ± 0.47	4.80 ± 0.70	1.95 ± 0.43	95,491	<0.001 **	0.78
Mesomorphy	2.14 ± 0.74	4.54 ± 0.46	3.38 ± 0.51	4.70 ± 0.78	3.22 ± 0.44	68,615	<0.001 **	0.71
Ectomorphy	4.8 ± 0.76	2.67 ± 0.34	3.24 ± 0.36	1.62 ± 0.90	4.64 ± 0.75	93,396	<0.001 **	0.77

** = *p* < 0.01.

**Table 4:** Descriptive statistics (mean ± SD) for the jumping and sprinting variables in participants by dominant somatotype.

Variables	Balanced Ectomorph (n = 25)	Balanced Mesomorph (n = 22)	Central (n = 24)	Mesomorph-Endomorph (n = 21)	Mesomorphic Ectomorph (n = 20)	F	*p*	η^2^
VJ (cm)	32.63 ± 8.45	35.71 ± 6.01	29.58 ± 7.53	27.61 ± 8.48	33.19 ± 5.64	4.043	0.004 **	0.13
PAPw (W)	2427.71 ± 828.56	2892.09 ± 575.76	2486.04 ±757.84	2726.33 ± 767.32	2403.21 ± 631.35	1.915	0.113	0.06
P:BM (W·Kg^−1^)	40.96 ± 9.53	45.06 ± 6.32	38.49 ± 7.57	37.59 ± 7.9	42.01 ± 7.29	3.134	0.018 *	0.10
ST (s)	4.26 ± 0.52	4.07 ± 0.29	4.47 ± 0.53	4.57 ± 0.7	4.24 ± 0.49	3.117	0.018 *	0.10
SV (m·s^−1^)	6.97 ± 0.76	7.41 ± 0.48	6.8 ± 0.77	6.69 ± 0.89	7.16 ± 0.73	3.306	0.014 *	0.11
Momentum (kg·m·s^−1^)	407.76 ± 94.41	502.47 ± 71.97	435.22 ± 98.3	482.84 ± 107.9	408.62 ± 96.05	4.657	0.002 **	0.14

* = *p* < 0.05; ** = *p* < 0.01; VJ = vertical jump; PAPw = peak anaerobic power measured in watts; P:BM = power to body mass ratio; ST = sprint time; SV = sprint velocity.

## Data Availability

Data is available for research purposes upon reasonable request to the corresponding author.

## References

[1] Carter J.L., Heath B.H. (1990). Somatotyping: Development and Applications.

[2] Carter J.E.L., Ackland T.R., Kerr D.A., Stapff A.B. (2005). Somatotype and size of elite female basketball players. J. Sports Sci..

[3] Heath B.H., Carter J.L. (1967). A modified somatotype method. Am. J. Phys. Anthropol..

[4] Fidelix Y.L., Berria J., Ferrari E.P., Ortiz J.G., Cetolin T., Petroski E.L. (2014). Somatotype of competitive youth soccer players from Brazil. J. Hum. Kinet..

[5] Guimarães Almeida L., Numata Filho E.S., dos Santos G.A., Carneiro Cardoso J.T., Rodrigues Moreira S. (2021). Anthropometric Profile and Functional Performance of Capoeira Competitors in the World Games. Int. J. Morphol..

[6] Laubach L.L., McConville J.T. (1969). The relationship of strength to body size and typology. Med. Sci. Sports Exerc..

[7] Marta C., Marinho D.A., Costa A.M., Barbosa T.M., Marques M.C. (2011). Somatotype is more interactive with strength than fat mass and physical activity in peripubertal children. J. Hum. Kinet..

[8] Lewandowska J., Buśko K., Pastuszak A., Boguszewska K. (2011). Somatotype variables related to muscle torque and power in judoists. J. Hum. Kinet..

[9] Kandel M., Baeyens J.P., Clarys P. (2014). Somatotype, training and performance in Ironman athletes. Eur. J. Sport Sci..

[10] Allen S.V., Hopkins W.G. (2015). Age of peak competitive performance of elite athletes: A systematic review. Sports Med..

[11] Ryan-Stewart H., Faulkner J., Jobson S. (2018). The influence of somatotype on anaerobic performance. PLoS ONE.

[12] Cinarli F.S., Kafkas M.E. (2019). The effect of somatotype characters on selected physical performance parameters. J. Phys. Educ. Stud..

[13] Marta C.C., Marinho D.A., Barbosa T.M., Carneiro A.L., Izquierdo M., Marques M.C. (2013). Effects of body fat and dominant somatotype on explosive strength and aerobic capacity trainability in prepubescent children. J. Strength Cond. Res..

[14] Kutseryb T., Vovkanych L., Hrynkiv M., Majevska S., Muzyka F. (2017). Peculiarities of the somatotype of athletes with different directions of the training process. J. Phys. Educ. Sport.

[15] Keogh J.W., Hume P.A., Pearson S.N., Mellow P. (2007). Anthropometric dimensions of male powerlifters of varying body mass. J. Sports Sci..

[16] Perez-Gomez J., Rodriguez G.V., Ara I., Olmedillas H., Chavarren J., González-Henriquez J.J., Calbet J.A. (2008). Role of muscle mass on sprint performance: Gender differences?. Eur. J. Appl. Physiol..

[17] Moncef C., Said M., Olfa N., Dagbaji G. (2012). Influence of morphological characteristics on physical and physiological performances of tunisian elite male handball players. Asian J. Sports Med..

[18] Barbieri D., Zaccagni L., Babić V., Rakovac M., Mišigoj-Duraković M., Gualdi-Russo E. (2017). Body composition and size in sprint athletes. J. Sports Med. Phys. Fit..

[19] Stewart A., Marfell-Jones M., Olds T., de Ridder H. (2011). International Standards for Anthropometric Assessment.

[20] Wilczyński B., Hinca J., Ślęzak D., Zorena K. (2021). The relationship between dynamic balance and jumping tests among adolescent amateur rugby players: A preliminary study. Int. J. Environ. Res. Public. Health.

[21] Sayers S.P., Harackiewicz D.V., Harman E.A., Frykman P.N., Rosenstein M.T. (1999). Cross-validation of three jump power equations. Med. Sci. Sports. Exerc..

[22] Jalilvand F., Banoocy N.K., Rumpf M.C., Lockie R.G. (2019). Relationship between body mass, peak power, and power-to-body mass ratio on sprint velocity and momentum in high-school football players. J. Strength Cond. Res..

[23] Gabbett T.J., Kelly J.N., Sheppard J.M. (2008). Speed, change of direction speed, and reactive agility of rugby league players. J. Strength Cond. Res..

[24] Mann J.B., Mayhew J.L., Dos Santos M.L., Dawes J.J., Signorile J.F. (2022). Momentum, Rather Than Velocity, Is a More Effective Measure of Improvements in Division IA Football Player Performance. J. Strength Cond. Res..

[25] Cohen J. (1988). Statistical Power Analysis for the Behavioral Sciences.

[26] Hopkins W.G. (2004). How to interpret changes in an athletic performance test. Sport Sci..

[27] Vila H., Manchado C., Rodriguez N., Abraldes J.A., Alcaraz P.E., Ferragut C. (2012). Anthropometric profile, vertical jump, and throwing velocity in elite female handball players by playing positions. J. Strength Cond. Res..

[28] Saha S. (2014). Somatotype, body composition and explosive power of athlete and non-athlete. J. Sports Med. Doping Stud..

[29] Esco M.R., Fedewa M.V., Cicone Z.S., Sinelnikov O.A., Sekulic D., Holmes C.J. (2018). Field-based performance tests are related to body fat percentage and fat-free mass, but not body mass index, in youth soccer players. Sports.

[30] Nikolaidis P.T. (2013). Body mass index and body fat percentage are associated with decreased physical fitness in adolescent and adult female volleyball players. J. Res. Med. Sci..

[31] Zary J.C., Reis V.M., Rouboa A., Silva A.J., Fernandes P.R. (2010). The somatotype and dermatoglyphic profiles of adult, junior and juvenile male Brazilian top-level volleyball players. Sci. Sports.

[32] Martín-Matillas M., Valadés D., Hernández-Hernández E., Olea-Serrano F., Sjöström M., Delgado-Fernández M., Ortega F.B. (2014). Anthropometric, body composition and somatotype characteristics of elite female volleyball players from the highest Spanish league. J. Sports Sci..

[33] Gualdi-Russo E., Zaccagni L. (2001). Somatotype, role and performance in elite volleyball players. J Sports Med. Phys. Fit..

[34] Aerenhouts D., Delecluse C., Hagman F., Taeymans J., Debaere S., Van Gheluwe B., Clarys P. (2012). Comparison of anthropometric characteristics and sprint start performance between elite adolescent and adult sprint athletes. Eur. J. Sport Sci..

[35] Rahmawati N.T., Budiharjo S., Ashizawa K. (2007). Somatotypes of young male athletes and non-athlete students in Yogyakarta, Indonesia. Anthropol. Sci..

[36] Abe T., Kawamoto K., Dankel S.J., Bell Z.W., Spitz R.W., Wong V., Loenneke J.P. (2020). Longitudinal associations between changes in body composition and changes in sprint performance in elite female sprinters. Eur. J. Sport Sci..

[37] Hansen K.T., Cronin J.B., Pickering S.L., Douglas L. (2011). Do force–time and power–time measures in a loaded jump squat differentiate between speed performance and playing level in elite and elite junior rugby union players?. J. Strength Cond. Res..

[38] Barr M.J., Sheppard J.M., Gabbett T.J., Newton R.U. (2014). Long-term training-induced changes in sprinting speed and sprint momentum in elite rugby union players. J. Strength Cond. Res..

[39] Baker D.G., Newton R.U. (2008). Comparison of lower body strength, power, acceleration, speed, agility, and sprint momentum to describe and compare playing rank among professional rugby league players. J. Strength Cond. Res..

